# Laser Welding of Fiber and Quartz Glass Ferrule

**DOI:** 10.3390/mi14050939

**Published:** 2023-04-26

**Authors:** Wenhua Wang

**Affiliations:** School of Electronic and Information Engineering, Guangdong Ocean University, Zhanjiang 524088, China; wangwh@gdou.edu.cn

**Keywords:** laser welding, quartz glass, optical fiber, CO_2_ laser

## Abstract

Optical fiber sensors fabricated by bonding have several limitations. To address these limitations, a CO_2_ laser welding process for an optical fiber and quartz glass ferrule is proposed in this study. A deep penetration welding method with optimal penetration (penetrating the base material only) is presented to weld a workpiece according to the requirements of the optical fiber light transmission, size characteristics of the optical fiber, and the keyhole effect of the deep penetration laser welding. Moreover, the influence of laser action time on the keyhole penetration is studied. Finally, laser welding is performed with a frequency of 24 kHz, power of 60 W, and duty cycle of 80% for 0.9 s. Subsequently, the optical fiber is subjected to out-of-focus annealing (0.83 mm, 20% duty cycle). The results show that deep penetration welding produces a perfect welding spot and has good quality; the hole generated from deep penetration welding has a smooth surface; the fiber can bear a maximum tensile force of 1.766 N. The performance of the optical fiber sensor is stable, and the maximum pressure deviation corresponding to the cavity length fluctuation is about 7.2 Pa. Additionally, the linear correlation coefficient R of the sensor is 0.99998.

## 1. Introduction

Laser welding is a non-contact heating welding process, which has the advantages of high output energy density, high machining accuracy, a small thermal action area, and low residual stress and produces [1,2,3,4]. It has a wide application prospect in the fields of intelligent detection and monitoring sensor systems, the aerospace industry, and high-tech photoelectric products [5,6,7].

Glass is a brittle material. Because of its excellent physical and chemical properties, it is widely used in optical sensing, electronic and micro-optical systems, and other fields. Optical fiber sensing systems are particularly important. They are used in the aerospace [8], civil engineering [9], petrochemical [10], and biomedical [11] fields because of their unique advantages. The components of optical fiber sensors must usually be firmly connected on a small scale for use in devices or systems that are deployed in harsh environments. Because optical fiber is composed of quartz glass, quartz glass is preferred over SiC [12] and other materials for designing optical fiber sensors with excellent performance [13]. The connection between glasses is a key problem; it has an important impact on the performance and environment of the sensor. Common glass connections include mechanical assembly, bonding, photo-adhesive bonding, and laser welding [1,2,3,14,15]. Mechanical assembly can easily lead to uneven stress and breaking of the glass; the bonding is significantly affected by adhesion, and it has poor stability. Thermal expansion and thermal degradation of the adhesive shorten the lifetime of the sensor and can even render it ineffective. The internal stress is uneven during the bonding process, resulting in cracks in thin components. The laser welding of glass is a desirable bonding method; however, researchers must still investigate how to obtain a better welding effect [1,2,3,16].

In these words, a CO_2_ laser is used to study the laser welding technology of quartz glass. The quartz glass fiber and quartz glass ferrule of an optical fiber sensor is firmly connected by laser welding. Laser welding does not affect the waveguide characteristics of the fiber and provides a technical guarantee for the good performance of optical fiber sensors. Furthermore, the operating environment is not limited by temperature and other problems, as in the case of sensors assembled by bonding [14].

## 2. Experiment

**Figure 1:**
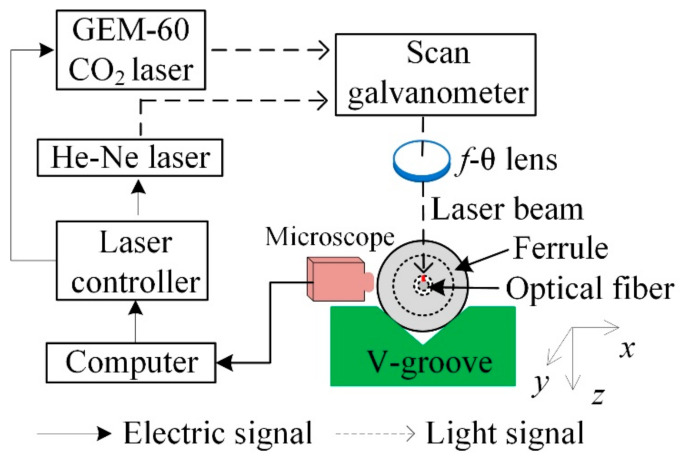
Deep penetration laser welding setup for optical fiber.

The experimental setup used for deep penetration laser welding (single-point welding or double-point welding) is shown in Figure 1. The laser is a waveguide cavity-sealed CO_2_ laser (Coherent Inc., GEM-60, Santa Clara, CA, USA) with a wavelength of 1064 nm and a continuous rated output power of 60 W. The unique resonator structure leads to good laser spot quality, with M^2^ = 1.1 and power stability of less than ±3%. For this laser, a control system based on the Labview platform was designed with a single-chip microcomputer as the core, which is used to accurately control the output time, duty cycle, and other parameters of the laser with a frequency of 24 kHz. The microscope in the figure is used to enlarge the image of the welding area to observe the laser welding effect during the welding process. The CO_2_ laser focuses its spot on the molten quartz glass ferrule through the focusing lens. The duration and average power of the laser are controlled by a software system based on the Labview platform. Reflector 1 is in the dotted line position when the CO_2_ laser works; a He-Ne laser, as the light source indicating the optical path, is used to adjust the optical path and determine the focal spot position. In the process of deep penetration laser welding, the laser focus is located on the surface of quartz glass ferrule just above the optical fiber. Therefore, after welding, the optical fiber is subject to out-of-focus annealing. Reflector 1 is in the solid line position when the He-Ne laser works; in the process of deep penetration laser welding, the laser beam, quartz glass ferrule, and quartz optical fiber are all stationary. Thus, it is considered that the effective thermal action of the laser can penetrate the wall of the glass ferrule by approximately 0.83 mm; however, the optical fiber does not fuse, owing to the thermal action of the laser. The quartz glass ferrule is fixed on a V-shaped groove, and the supporting platform of the V-shaped groove can be adjusted in the X, Y, and Z directions.

**Figure 2:**
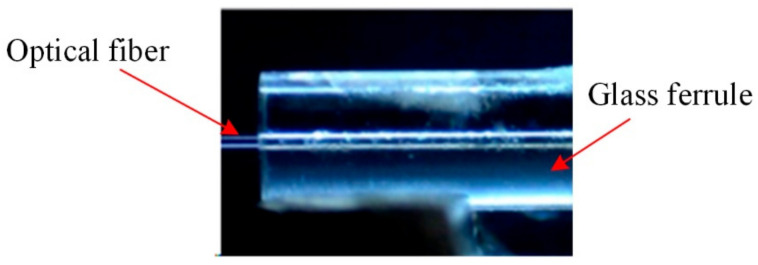
Workpiece to be welded: optical fiber is inserted into the through hole of the quartz glass ferrule.

**Figure 3:**
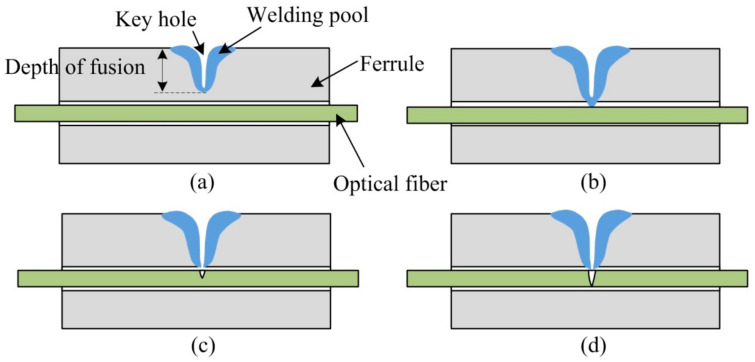
Schematic of the effect of deep penetration welding between the optical fiber and quartz glass ferrule. (**a**) Incomplete penetration: insufficient laser energy and keyhole penetration depth that is too small; (**b**) optimal penetration (penetrating the base material only): proper laser energy and appropriate penetration depth of keyhole; (**c**) moderate penetration: sufficient laser energy and proper penetration depth of keyhole; and (**d**) over penetration: excessive laser energy, and keyhole penetration depth that is too large.

The quartz glass ferrule used in the experiment has a length of 7 mm and diameter of 1.8 mm. There is a through hole with a diameter of 126–128 µm in the axial position of the ferrule, and one end of the through hole has a tapered opening for forming the cavity of the optical fiber sensor. Before laser welding, the optical fiber was inserted into the through hole. The diameter of the optical fiber is 125 µm. Before inserting the optical fiber into the through hole of the quartz glass ferrule, the ferrule was cleaned three times by ultrasonic cleaning and dried, and the optical fiber was wiped with alcohol after removing the coating layer. The effect of inserting the optical fiber into the through hole is shown in Figure 2. There was a very small gap between the optical fiber and quartz glass ferrule. The schematic diagram of the welding effect with different parameters is shown in Figure 3. A keyhole was formed on the wall of the quartz glass ferrule.

The keyhole is formed because quartz glass has a high coefficient of CO_2_ laser absorption, which leads to the rapid rise of temperature at the glass surface and heat transmission to the inside of the material. In addition to the temperature rise, melting, and gasification, a large amount of material steam is formed at the same time. The expansion pressure of the steam bends the glass surface in the molten state downwards and causes pits. The laser beam enters the glass surface at the bottom of the pits through the steam, resulting in a new process of temperature rise, melting, gasification, and downward glass surface bending. Therefore, under the continuous action of laser energy, the pits are deepened to form an hourglass-shaped (inverted cone-shaped) hole, which is surrounded by a molten pool.

After studying the keyhole and molten pool, researchers suggest that the deep penetration laser welding process has four states: incomplete, optimal (penetrating the base material only), moderate, and over penetration [17]. Among them, the optimal penetration state means that after the keyhole is formed by laser welding, the keyhole is close to the lower surface of the workpiece but does not penetrate it, while the liquid material at the bottom of the keyhole can penetrate through the back surface of the workpiece, resulting in the melting of the back surface. However, the back surface of the workpiece cannot form a large melting width, resulting in unreliable penetration and weak welding. Moderate penetration means that the hole just penetrates the workpiece. After the hole penetrates the workpiece, the material vapor oozes below the workpiece along the hole. The generated recoil pressure causes the width of the back of the molten pool to be greatly increased by the liquid material, forming a larger melting width and firm weld. For most deep-penetration laser welding, moderate penetration is the most ideal. At this time, the keyhole penetrates the workpiece to ensure complete penetration, while the weld pool is not too wide to sag the surface of the weld. This benchmark can be used as the benchmark of penetration detection during deep penetration laser welding, which is applicable to both metal and non-metallic materials such as glass. However, for the deep penetration welding between the fused quartz collimator capillary and optical fiber in this paper, if the proper penetration state is adopted as the welding benchmark, the waveguide characteristics of the optical fiber will be easily destroyed, and the optical fiber will be blown off at the moment when the keyhole just penetrates the molten pool, thus causing the failure of deep penetration welding. Therefore, the parameters must be controlled so that deep penetration welding is the only penetration state. At this time, the hole is close to the inner wall of the capillary but does not penetrate the capillary wall. However, the molten quartz at the bottom of the hole is in a molten state, and the molten quartz in the molten state moves toward the optical fiber under the action of surface tension and gravity, thus squeezing and sticking the optical fiber. As the optical fiber is bound in the narrow capillary, the optimal penetration is suitable for the deep penetration welding in this study, which not only realizes the welding between the capillary and optical fiber but also ensures that the optical fiber will not be fused, as shown in Figure 3b. However, for moderate and over penetration shown in Figure 3c,d, when the hole just penetrates the capillary wall, the molten quartz ejected into the capillary along the bottom of the hole destroys the waveguide characteristics of the optical fiber and can even fuse the optical fiber.

**Figure 4:**
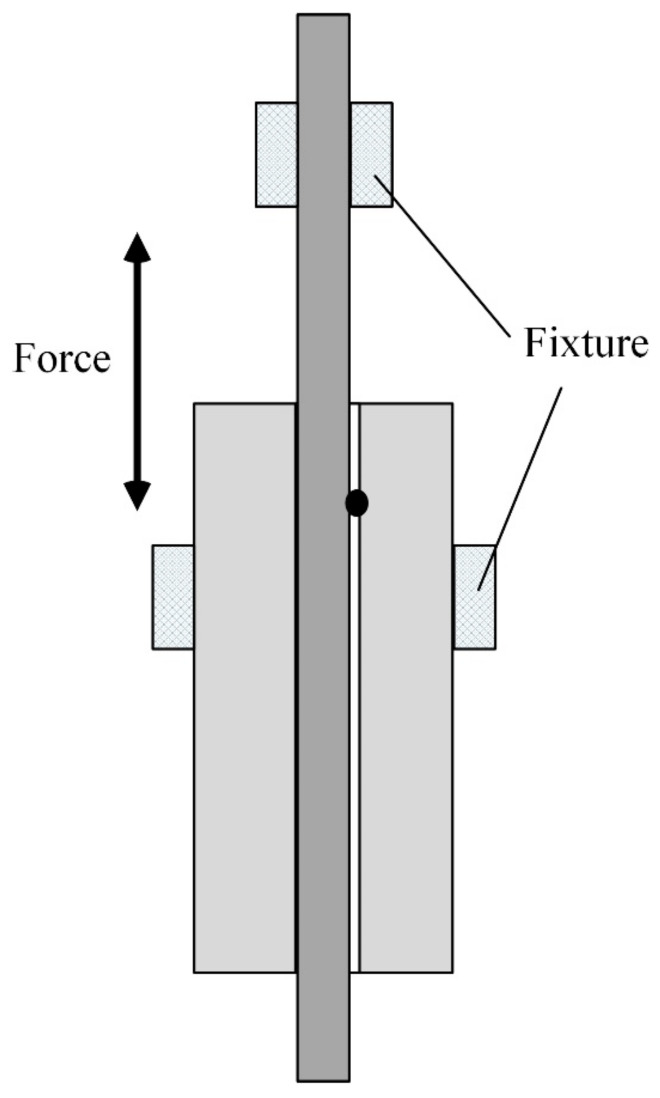
Schematic of tensile test of the welded samples.

After welding, the macroscopic and microscopic morphologies are observed using a microscope. A tensile test of the welded sample is conducted, and the performance of the welded sensor is tested. The schematic diagram of the tensile test of the welded samples is shown in Figure 4.

## 3. Results and Analysis

**Figure 5:**
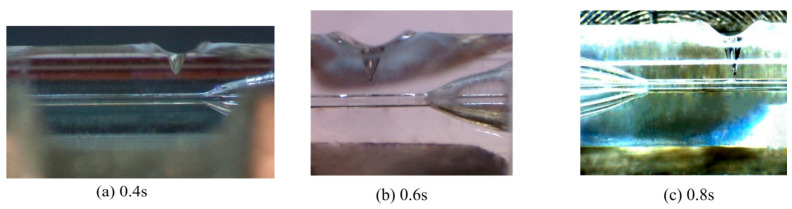
Welding results of different laser action times.

**Figure 6:**
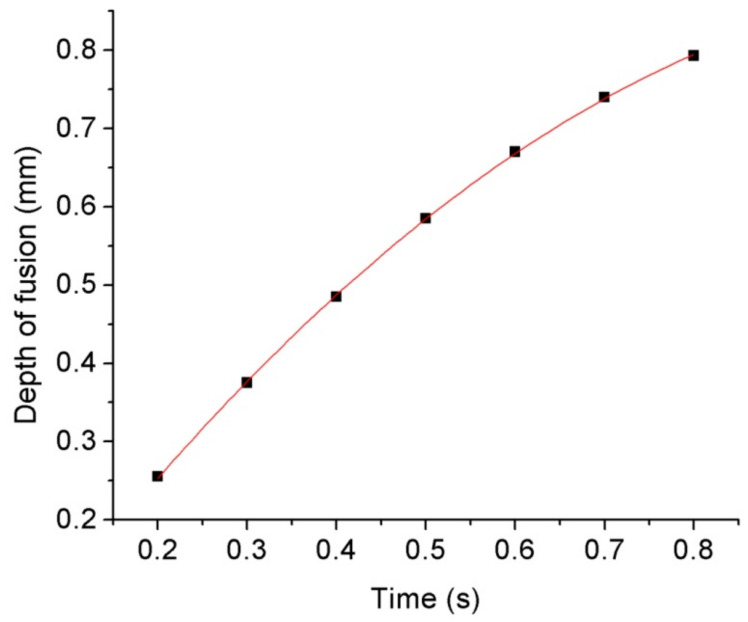
Relationship between laser action time and penetration depth of the keyhole.

As the deep penetration welding between the quartz glass ferrule and optical fiber requires long-term laser action or a high-power laser to reach the required penetration depth, and the laser power used in the experiment is 60 W, the duty cycle of 80% is adopted to maintain the same; then, the effect of laser action time on the penetration depth of the keyhole during deep penetration welding is studied. Deep penetration laser welding was conducted for 0.2, 0.3, 0.4, 0.5, 0.6, 0.7, and 0.8 s. Because of the keyhole effect of deep penetration laser welding, an inverted cone-shaped keyhole is formed on the wall of the glass ferrule. The results are shown in Figure 5. As shown in Figure 5, the molten quartz glass at the edge of the keyhole is convex, which is because the molten quartz glass in the molten pool has certain fluidity during the deep penetration welding process, and the keyhole is formed under the pressure of a laser beam and steam in the keyhole. The molten quartz in the molten state in the molten pool around the keyhole flows outward at a relatively slow speed under the action of convection. The shape formed by cooling and solidification after stopping the laser action and the shape of the keyhole affects the transmission of laser energy. As the laser generates multiple Fresnel reflections, as shown in Figure 3 on the hole wall, the laser energy is transmitted to the bottom of the hole and absorbed, which then causes the material to heat up, melt, and vaporize. Based on this, the penetration of the hole deepens with the extension of the laser action time. The relationship between the laser action time and penetration depth of the keyhole is shown in Figure 6. The relationship between the penetration depth *z* and laser action time *t* is obtained by polynomial fitting as follows:

(1) $$z=-0.67t^{2}+1.576t-0.04$$

The correlation coefficient is 0.99995. 

**Figure 7:**
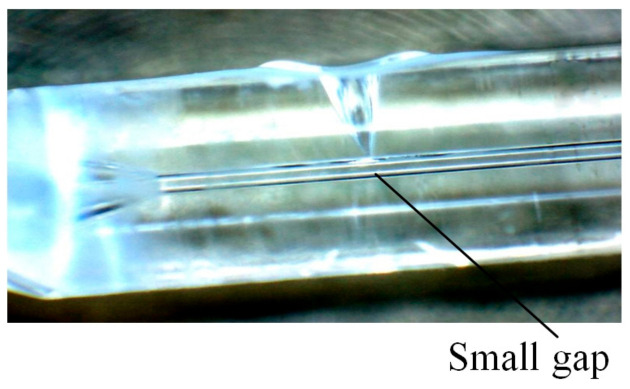
Result of optimal deep penetration welding.

As shown by the above formula, when the laser action time is 0.9 s, the penetration depth is 0.836 mm, and the diameter of the through hole of the glass ferrule is 126–128 μm. The middle value of 127 μm is taken for calculation, and the thickness of the pipe wall is 0.9 − 0.127/2 ≈ 0.837 mm. Therefore, to ensure that the optical fiber is not fused by the heat generated by the laser, the laser action time of 0.9 s is adopted for deep penetration welding. In addition, since quartz glass must be heated to 1750 °C or higher when welding, the welding area of the optical fiber must be annealed after deep penetration welding, and the total time of laser action is 1.2 s. The period of 0–0.9 s is the deep penetration welding process of the laser, with a duty cycle of 80%; the period of 0.9–1.2 s is the laser annealing process of the optical fiber in the welding area, with a duty cycle of 20%. The welding results are shown in Figure 7. The results show that the required penetration depth can be obtained by increasing the laser action time. When the laser action time is 0.9 s, the inverted cone-shaped keyhole can achieve the optimal penetration state described in Part 3, and the optical fiber is fixed on the inner wall of the quartz glass ferrule: the glass in the molten state at the bottom of the keyhole tends to drip under the action of surface tension, gravity, and laser beam light pressure and moves towards the lower optical fiber. Then, the optical fiber is pressed down to the inner wall of the glass ferrule, so that it is stuck and pressed into the tiny glass ferrule through the hole. Finally, the deep penetration point welding between the quartz glass ferrule and the optical fiber is realized.

**Figure 8:**
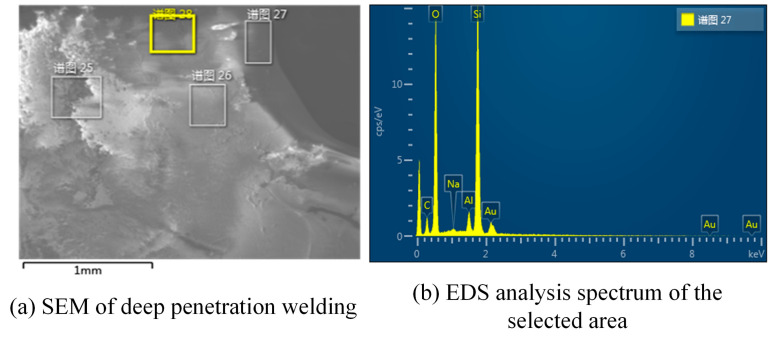
SEM and EDS analyses of the top view of the keyhole in deep penetration welding; the meaning of Chinese characters in the picture is selection area of spectrogram.

**Figure 9:**
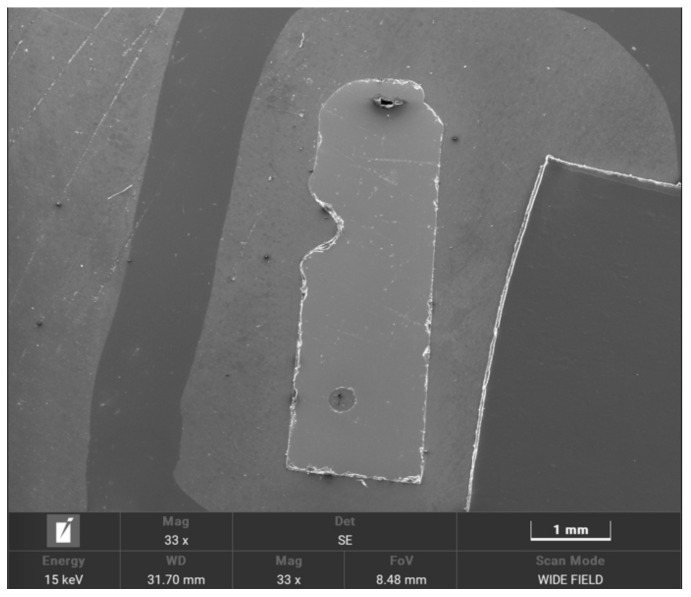
SEM image of the keyhole section formed by deep penetration welding.

The SEM and EDS analyses of the top view of the keyhole in deep penetration welding are shown in Figure 8. Figure 8a shows that the surface of the hole formed by the molten pool is smooth, which helps the laser energy to form the Fresnel reflection, and then transfer to the bottom of the hole, resulting in improving the welding efficiency and quality. The EDS analysis results are shown in Table 1. The EDS analysis in Table 1 and Figure 8b shows that the main components of deep penetration welding materials are O and Si. Since the workpiece may be polluted and the quartz glass may be partially carbonized during deep penetration welding, a certain proportion of C is present in the EDS analysis results. The glass ferrule, after deep penetration welding, is fixed on the colloid, and then its side is ground and polished until a part of the hole formed by deep penetration welding is ground off. Then, the cross section of the hole is examined by SEM, and the results are shown in Figure 9. The SEM shows the entire morphological features of the ferrule after grinding on the side, and the V-shaped area is the part of the keyhole after grinding on the side. As shown by the figure, the shape of the hole conforms to the tapered hole formed by laser deep penetration welding, and the surface of the hole is not as smooth as that shown in Figure 9. This may be because there are a few cracks formed under the action of external forces during grinding and polishing because the molten pool has residual stress during cooling and solidification. The cracks do not affect the actual appearance of the inherent hole of the sensor or the application of the sensor. The key of the welding workpiece is that the waveguide characteristics of the optical fiber are not affected, and the optical fiber on the inner wall of the glass ferrule has sufficient bonding strength. In addition, the rough surface of the hole shown in Figure 9 may be caused by grinding and polishing, or the powder that remains after grinding and polishing. Additionally, the original smooth surface of the glass ferrule also became serrated due to grinding and polishing.

**Figure 10:**
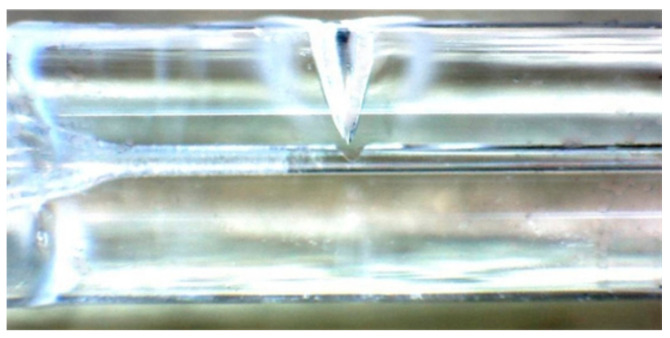
Welding with excessive laser energy leads to the destruction of the waveguide characteristics of optical fiber.

As described later, the deep penetration welding between the optical fiber and glass ferrule should be optimal rather than moderate. Figure 10 shows the results of deep penetration welding when the duty cycle is 80% and the laser action time is 0.95 s. The figure shows that the penetration depth with a welding duration of 0.95 s is just equal to the wall thickness of the glass ferrule, which belongs to moderate penetration. However, at this time, the molten quartz flowing out of the keyhole and the laser action at the moment of welding almost fuse the optical fiber. Therefore, the welding of optical fiber is different from deep penetration laser welding in other situations. Because of the uniqueness of optical fiber, the deep penetration welding of optical fiber and the wall of quartz glass ferrule can only be performed by optimal penetration.

**Figure 11:**
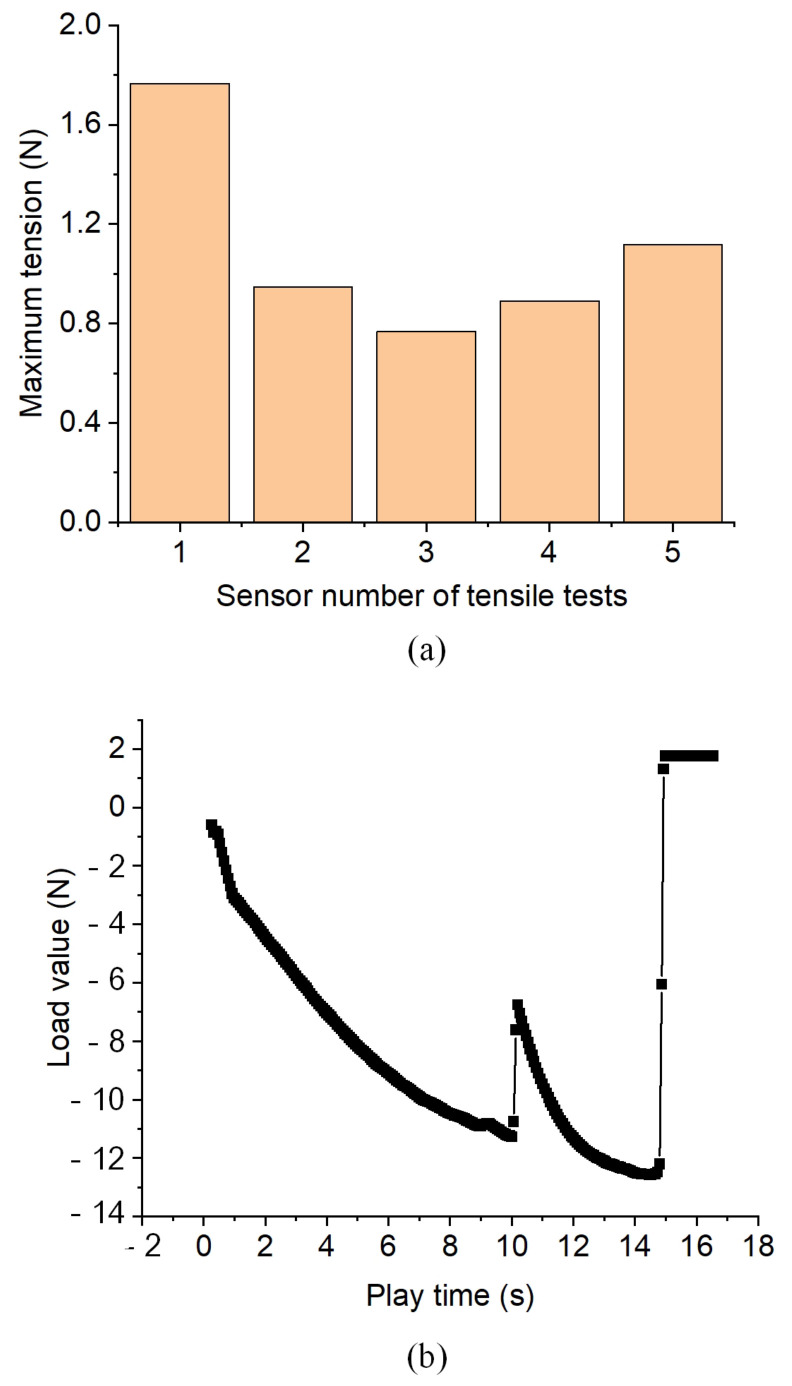
Tensile test results of welded components: (**a**) the maximum force of 5 samples when stretching; (**b**) the stretching process of the sample with the maximum tension.

**Figure 12:**
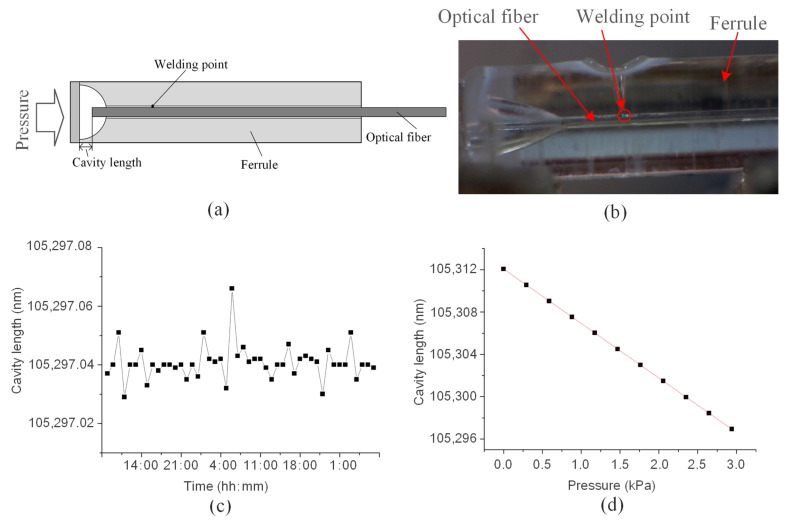
Performance test results of a laser welding sensor: (**a**,**b**) structure diagram of the sensor; (**c**) cavity length stability of the sensor; and (**d**) pressure response characteristics of the sensor.

The tensile test of the welded components is carried out as shown in Figure 4, and the test results are shown in Figure 11. Figure 11a shows that after the connection between the quartz glass ferrule and the optical fiber is welded, the maximum tensile force that the optical fiber can bear is 1.766 N, and the minimum tensile force is 0.7669 N. Figure 11b shows the tensile process of one of the weldments. Although the optical fiber can bear a small tensile force, it is packaged after welding to avoid harming the usability of the welded products. In addition, there is a certain difference in the maximum tensile force that can be borne by the welded product. This difference is aroused because the size of quartz glass ferrule is not standard and has relatively large errors, and the tensile force that can be withstood by optical fiber is different when optimal penetration is adopted. After the sensor structure is welded, as shown in Figure 12a, the packaged sensor is placed in the actual water environment for testing for 48 h. The cavity length of the sensor (the cavity length is an important parameter of the sensor, and the change in cavity length is an important parameter for the sensor to obtain the change in external environmental parameters) is relatively stable, and the maximum pressure deviation corresponding to cavity length fluctuation is about 7.2 Pa, as shown in Figure 12b; the pressure response characteristics of the sensor are shown in Figure 12c. When the cavity length is affected by the environmental pressure, the sensitivity is 5.15 nm/kPa, which has good linearity. The correlation coefficient is 0.99988. Therefore, the deep penetration welding of quartz glass ferrule and optical fiber using a CO_2_ laser proposed in this study was successful. This welding technique can improve the performance of bonding optical fiber sensors.

## 4. Conclusions

In this study, a CO_2_ laser was used to rapidly heat and melt quartz glass according to the characteristics of the absorption of light wavelengths by quartz glass. The heat accumulation of the glass softened and vaporized the quartz glass, forming an inverted cone-shaped hole on the wall of the glass ferrule to realize deep fusion welding between the quartz glass ferrule and optical fiber. Considering the hairlike optical fiber and its waveguide characteristics, deep penetration welding in the optimal penetration state is proposed to ensure the effect of deep penetration welding and retain the waveguide characteristics of the optical fiber. According to the size of the glass ferrule used in this study, deep penetration welding was performed at a frequency of 24 kHz and power of 60 W for 0.9 s. Microscopy results show that the welding spot was perfect, and the welding quality was good. For the well-welded optical fiber sensor, the keyhole generated from deep penetration welding has a smooth surface. The optical fiber can bear a maximum tensile force of 1.9 N, and the sensor has a cavity length stability of 0.2 nm and a resolution of 0.086 nm.

## Figures and Tables

**Table 1:** EDS analysis.

Element	Apparent Concentration	wt%	Atomic Percent
C	0.44	17.28	24.68
O	9.79	53.66	57.53
Al	0.33	1.77	1.13
Si	5.00	27.28	16.66
Total		100.00	100.00

## Data Availability

Not applicable.
